# Fast track antibody V-gene rescue, recombinant expression in plants and characterization of a *Pf*MSP4-specific antibody

**DOI:** 10.1186/s12936-015-0577-7

**Published:** 2015-02-05

**Authors:** Stephanie Kapelski, Alexander Boes, Holger Spiegel, Melanie de Almeida, Torsten Klockenbring, Andreas Reimann, Rainer Fischer, Stefan Barth, Rolf Fendel

**Affiliations:** Fraunhofer Institute for Molecular Biology and Applied Ecology IME, Forckenbeckstraße 6, 52074 Aachen, Germany; RWTH Aachen University, Institute for Molecular Biotechnology, Worringer Weg 1, 52074 Aachen, Germany; Institute for Applied Medical Engineering at RWTH Aachen University and Hospital, Department of Experimental Medicine and Immunotherapy, Pauwelsstraße 20, 52074 Aachen, Germany

**Keywords:** Antibody characterization, Hybridoma antibody cloning, *Plasmodium falciparum*, Transient antibody expression, Plant-based production, Vacuum infiltration, *Nicotiana benthamiana*

## Abstract

**Background:**

Monoclonal antibodies (mAbs) are essential tools in biological research, diagnosis and therapy, and are conventionally produced in murine hybridoma cell lines. Professional applications of mAbs depend on the steady supply of material. Because hybridoma cultures can stop producing the antibody or even die, preservation of the unique epitope specificity of mAbs by rescue of the sequences encoding the antibody variable domains (V regions) is important. The availability of these sequences enables not only the recombinant expression of the original antibody for further applications, but opens the road for antibody engineering towards innovative diagnostic or therapeutic applications. A time- and cost-efficient production system enabling the detailed analysis of the antibodies is an essential requirement in this context.

**Methods:**

Sequences were rescued from three hybridoma cell lines, subjected to sequence analysis, subcloned into binary expression vectors and recombinantly expressed as chimeric mAb (constant regions of human IgG1:k1) in *Nicotiana benthamiana* plants. The properties of the recombinant and the murine mAbs were compared using competition enzyme-linked immunosorbent assay (ELISA) and surface plasmon resonance (SPR) spectroscopy. The recognition of native *Pf*MSP4 by the recombinant mAb was analysed by immunofluorescence staining of *Pf* 3D7A schizonts and by western blot analysis of merozoite extract.

**Results:**

The rescued sequences of all three hybridoma cell lines were identical. The recombinant mAb was successfully expressed as IgG in plants at moderate levels (45 mg/kg fresh leaf weight). Preservation of the original epitope was demonstrated in a competition ELISA, using recombinant mAb and the three murine mAbs. EGF_*Pf*MSP4-specific affinities were determined by SPR spectroscopy to 8 nM and 10 nM for the murine or recombinant mAb, respectively. Binding to parasite *Pf*MSP4 was confirmed in an immunofluorescence assay showing a characteristic staining pattern and by western blot analysis using merozoite extract.

**Conclusions:**

As demonstrated by the example of an EGF_*Pf*MSP4-specific antibody, the described combination of a simple and efficient hybridoma antibody cloning approach with the flexible, robust and cost-efficient transient expression system suitable to rapidly produce mg-amounts of functional recombinant antibodies provides an attractive method for the generation of mAbs and their derivatives as research tool, novel therapeutics or diagnostics.

## Background

Monoclonal antibodies (mAbs) have become irreplaceable for a wide variety of applications, including laboratory analytics, diagnosis of chronic and infectious diseases as well as targeted therapy [1]. All this was made possible by the development of the hybridoma technology by Köhler and Milstein in 1975 [2]. However, hybridoma cultures can become unstable over time, and antibody expression declines or ceases all together [3,4], meaning that the information encoded in the variable regions (V regions) must be rescued to preserve a given antibody’s unique epitope specificity and affinity. Based on the rescued V region information various recombinant antibody formats can be generated, including full-size recombinant (chimeric) antibodies, minibodies, Fab fragments, and single-chain variable fragments (scFv) [4,5]. In the field of malaria, most of these formats have been introduced as potential therapeutic molecules [6]. Two predominant applications are envisaged in this review: The development of bispecific scFvs or Fab fragments for the recruitment of immune effector cells, or the generation of several mAb-isotypes (IgG, IgA, IgM) to specifically modify effector functions. This can only be facilitated through exact knowledge of the sequence information. Furthermore, the V regions can also be engineered, e.g., humanized by grafting the complementarity determining regions (CDRs) onto suitable human variable domain framework regions (FWRs) followed by affinity maturation, thus avoiding the drawbacks of human anti-mouse immune responses in therapeutic applications [7]. To efficiently match the complete variability of the V-region repertoire, many primer sets used for their rescue contain nucleotide degeneracies or consensus sequences [8-11]. In combination with mutations that can occur during the inevitable PCR-amplification steps, this can lead to deviations of the nucleotide-, and eventually amino acid sequences between the parental and the rescued antibody sequence. As a result changes or losses of epitope specificity and/or affinity as well as insolubility or extremely low expression levels have been observed [12]. Careful analysis of the rescued V-region sequences must be performed to identify and subsequently remove such unwanted mutations. In many cases it will be impossible to distinguish mutations accidentally introduced by the rescue procedure from those occurring in mAbs as a result of affinity maturation. To verify the obtained sequences, the antibody has to be expressed recombinantly and analysed for antigen binding in comparative assays involving the parental murine mAb. For this purpose, transient expression of recombinant mAbs in plants is a cost-efficient and robust method [13].

By the example of a murine mAb specific for the *Plasmodium falciparum* merozoite surface protein 4 (*Pf*MSP4), a workflow was developed, which allows the rapid, inexpensive and accurate rescue and functional confirmation of V-region sequences. *Pf*MSP4 is one of several glycophosphatidylinositol (GPI)-anchored surface proteins expressed at the merozoite stage and it has been identified as a potential malaria vaccine, although it has not reached clinical development. Nevertheless, promising data have confirmed its protective role in murine mouse models. Multiple studies have shown that vaccination with a combination of MSP4 and MSP5 protects mice against both homologous and heterologous challenge [14,15]. This work describes the hybridoma antibody cloning of the heavy and kappa light chain V-region sequences (V_H_/V_L(κ)_) of a novel murine antibody specific for the epidermal growth factor (EGF)-like domain of *Pf*MSP4 (EGF_*Pf*MSP4), the cloning of the respective V-region sequences into a plant expression vector containing a human IgG1 Fc or kappa1 domain, as well as the transient expression in tobacco and detailed characterization of the recombinant mAb.

## Methods

### Generation of monoclonal hybridoma cell lines specific for EGF_*Pf*MSP4

**Figure 1:**
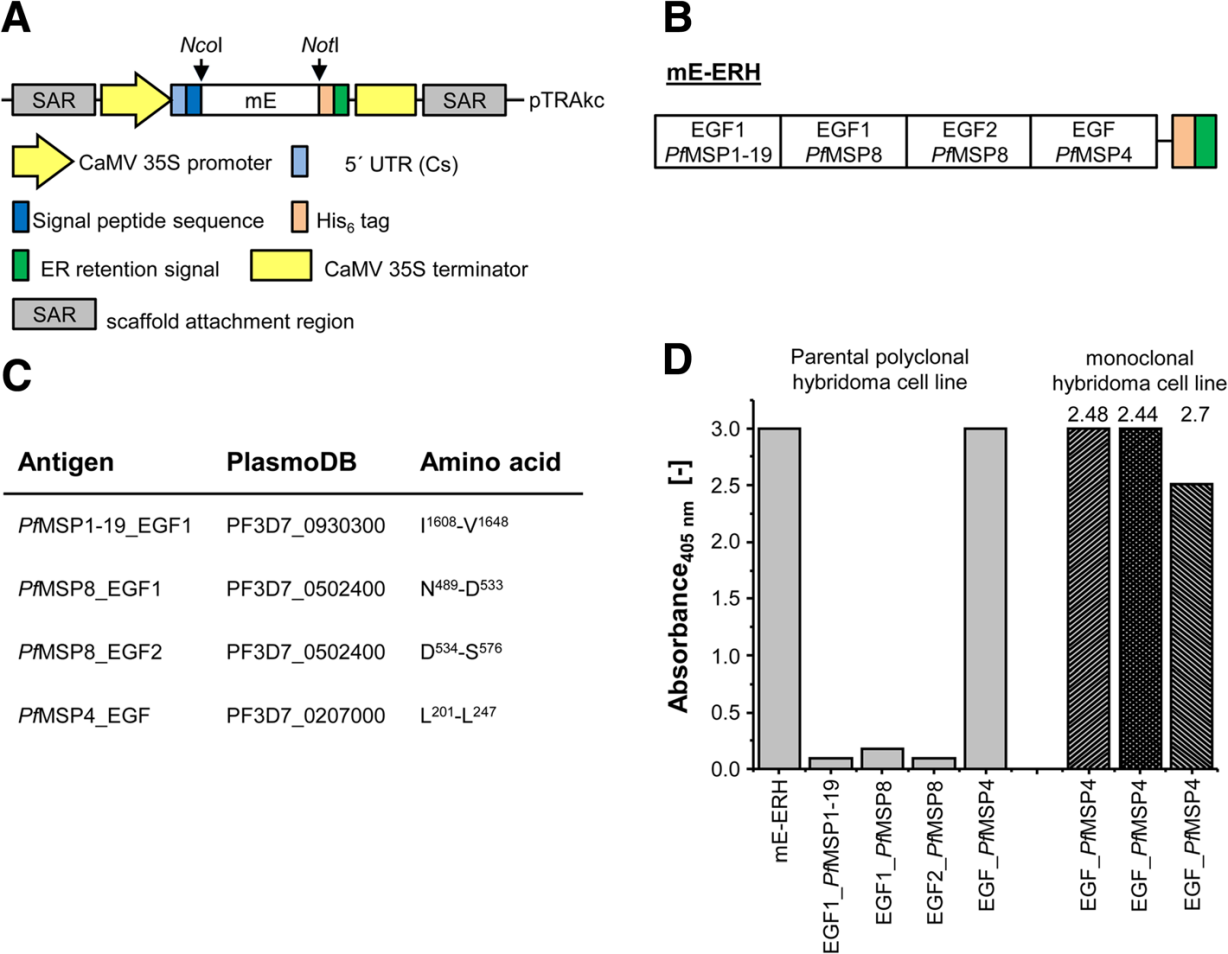
**Generation of mE-ERH and isolation of EGF_** ***Pf*** **MSP4-specific monoclonal hybridoma cell lines. (A)** Expression cassette of the pTRAkc_mE-ERH vector used for the expression of mE-ERH. The vector contains the *Cauliflower mosaic virus* 35S promoter (CaMV 35S promoter), a 5′ UTR of the Chalcone synthase of *Petroselium crispum* (5′ UTR (Cs)), a signal peptide sequence followed by mE (the multi-EGF_MSP protein as explained in **(B)** and **(C)**), which was inserted using *Nco*I/*Not*I restriction sites, a 6-fold histidine-tag for purification, an ER-retention signal and the CaMV 35S terminator flank by scaffold attachment regions (SAR). **(B)** Components of the mE-ERH multidomain fusion protein. The protein consists of the EGF-like domains of MSP1-19, both of MSP8, and MSP4. **(C)** Peptide-sequences of the EGF-like domains of the MSPs and corresponding PlasmoDB accession numbers. **(D)** Reactivity of the parental polyclonal hybridoma cell line towards mE-ERH and its subdomains. The three selected monoclonal hybridoma cell lines 2.48, 2.44 and 2.7 show exclusive reactivity towards EGF_*Pf*MSP4.

mE-ERH, a fusion protein comprising the EGF-like domains of MSP1-19, MSP8 and MSP4 (Figure 1A,B,C), was generated by genetic fusion of the corresponding protein domains, cloning into pTRAkc-ERH, expression as his-tagged and ER-retained protein by agroinfiltration of *Nicotiana benthamiana* leaves and purification essentially as described previously [16]. To determine the specificity of the raised antibodies, the EGF-like domains were separately fused C-terminally to the red fluorescent protein (DsRed) and expressed accordingly. Twenty-five μg of purified mE-ERH was mixed with GERBU MM and used for the immunization of BALB/c mice by one prime and six consecutive boosts at a 14-day interval. Hybridoma cell lines were finally generated by fusing mouse myeloma cells (cell line Sp2/0-Ag14, obtained from ATCC (CRL-1581)) to isolated spleen cells from these mE-ERH-immunized BALB/c mice. The animal experiments were approved by the Landesamt für Natur, Umwelt und Verbraucherschutz Nordrhein-Westfalen (LANUV), Germany, reference number 8.87.-51.05.30.10.077. All animals received humane care according to the requirements of the German Tierschutzgesetz, §8 Abs. 1 and the Guide for the Care and Use of Laboratory Animals published by the National Institutes of Health.

The screening ELISAs were performed by coating 50 ng of antigen (mE-ERH or the single EGFs as DsRed-fusions). After blocking with 5% skimmed milk, culture supernatant was applied. Bound antibodies were detected by a goat anti-mouse IgG (Fc-specific) conjugated to peroxidase (PO) (Jackson Immuno Research, West Grove, PA, USA) followed by visualization using ABTS (Roche, Mannheim, Germany) according to the manufacturer’s instructions. Absorbance was read at 405 nm. Plates were washed intensively with PBS-T between steps.

### Primers and vectors

The outer primer set for the initial isolation of the V regions (including V, D and J genes) was described by Tiller *et al.* [8]. The V_H_ amplification set consisted of one forward primer to amplify all V_H_ regions, which anneals in the FWR1 of the V_H_ region, thus accepting partial mispriming, and one reverse primer for each immunoglobulin subtype, which binds in the constant domain. The V_L(k)_ regions were amplified using primers annealing in the leader peptide sequence and in the constant domain. Therefore the entire V_L_ region, including V- and J-gene fragments, was readable after sequencing.

The pTRAkc-based [17] plant expression vectors, pTRAkt_HC and pTRAkt_LC were used for plant expression of recombinant chimeric mouse-human IgG1. These vectors contain the 5′ untranslated region (UTR) from *Tobacco etch virus* (TEV) instead of the corresponding region of the *Petroselinum crispum* chalcone synthase found in pTRAkc-mE-ERH. The expression cassette encodes a murine IgG leader sequence (GenBank ID DQ407610) providing a signal peptide for secretion of the recombinant protein, and harbouring the *Age*I restriction site used for the cloning of PCR-amplified V-region genes [8]. The pTRAkt-HC and pTRAkt-LC vectors also include sequences encoding the human constant domain for the heavy chain (hC_γ1_) allotype IgG1m17,1 (pTRAkt_HC) and the light chain (hC_κ1_) allotype Km3 (pTRAkt_LC) [18], respectively, featuring the *Sal*I (pTRAkt_HC) and the *BsiW*I (pTRAkt_LC) restriction sites for cloning.

### RNA isolation, cDNA synthesis and DNA amplification by PCR

Hybridoma cells were harvested and stored in RNA protect cell reagent (Qiagen, Hilden, Germany). RNA was isolated using the M&N NucleoSpin RNA II Kit (Macherey Nagel, Düren, Germany). First strand cDNA was synthesized with SuperScript® III reverse transcriptase (Invitrogen, Karlsruhe, Germany) using oligo-dT primers. V regions were amplified from this cDNA by PCR using the Expand High Fidelity PCR system (Roche) essentially as previously described although with only 30 amplification cycles [8]. All kits and reagents were used according to the manufacturers’ instructions.

### Sequence analysis, specific primer design and V-region cloning

The PCR products of the V_H_ and V_L_ regions were purified using the M&N NucleoSpin® Extract II Kit (Macherey-Nagel) and sequenced on an ABI PRISM® 3730 Genetic Analyzer (Applied Biosystems, Carlsbad, USA). Sequences were analysed using the IMGT/V-quest [19,20] and NCBI nucleotide BLAST [21] online tools. The FWR1 of the V_H_-gene sequence was aligned to the most probable germ-line sequence, which accordingly served as template for the design of a FWR1-specific V_H_-region forward cloning primer. This was necessary because the forward primer of the V_H_ region intends partial mispriming, as explained above. Because the reverse V_H_ and both V_L(k)_ primers anneal outside the V (D and J) regions, these were completely readable. Therefore, the corresponding cloning primers did not need sequence adjustment and could be directly derived from the sequences. Primers were designed to facilitate in-frame insertion of the amplified V regions into pTRAkt_HC or pTRAkt_LC by *Age*I and *Sal*I for the V_H_ region, or *Age*I and *BsiW*I for the V_L(k)_ region, respectively. After ligation, the plasmids were introduced into *Escherichia coli* strain DH5α for cloning and the sequences of isolated plasmids were confirmed as described above.

### Production of recombinant antibodies in plants

Full-length recombinant 2.44IgG1 was produced by infiltrating *Nicotiana benthamiana* plants with *Agrobacterium tumefaciens* strain GV3101 PMP90RK (GmR, KmR, RifR) [22]. pTRAkt_2.44HC and pTRAkt_2.44LC were separately transformed into electrocompetent *Agrobacterium tumefaciens* using a Multiporator (Eppendorf, Hamburg, Germany). An additional *Agrobacterium tumefaciens* strain containing pTRAkc-p19si [17] was used as a silencing inhibitor [23]. All three clones were grown separately and used for the infiltration of *Nicotiana benthamiana* plants in a ratio of 2:2:1 for bacterial strains containing pTRAkt_2.44HC, pTRAkt_2.44LC and pTRAkc_p19si, respectively, as previously described [17].

After five days, leaves were harvested and shred in 3 × (v/w) ice-cold extraction buffer (PBS containing 10 mM sodium disulphite, pH 8.0). The resulting extract was prefiltered through Miracloth (EMD Millipore, Darmstadt, Germany). A substantial fraction of contaminating plant proteins was precipitated using 500 mM sodium chloride at pH 8.0 and incubated for 30 min at 4°C before centrifugation at 38,000 × g for 20 min at 4°C. The supernatant was filtered through a glass-fibre prefilter (Sartorius Stedim, Goettingen, Germany) and a 0.45-μm filter (cellulose acetate, Sartorius Stedim). The 2.44IgG1 antibody was finally purified by MabSelect™ chromatography (GE Healthcare, Uppsala, Sweden) according to the manufacturer’s recommendations, with 0.2 M Tris–HCl (pH 9.0) as the binding and washing buffer, and 0.2 M sodium citrate (pH 2.7) as the elution buffer. Eluted mAbs were immediately neutralized using 1 M Tris–HCl (pH 9.0), concentrated using concentrating centrifugal devices (Vivaspin 30 kDa MWCO, Hydrosart membrane, Sartorius Stedim) and stored in RPMI1640 containing 25 mM HEPES at −20°C.

### Purification of murine IgGs from hybridoma culture supernatants

The three hybridoma clones producing antibodies specific for MSP4 (2.44, 2.48, 2.7) were cultivated in ISF-1 medium (Biochrom, Berlin, Germany). The antibodies were purified from 300 ml supernatant by MabSelect™ chromatography as described above.

### Analysis of recombinant antibody function

#### ELISA and competition ELISA

The binding of recombinant 2.44IgG1 was assessed by ELISA. Each well of a 96-well high-binding plate (Greiner Bio-One, Frickenhausen, Germany) was coated with 100 ng of antigen in PBS and blocked with 2% BSA in PBS. The IgGs were applied as 1:2 serial dilutions in duplicate. Bound 2.44IgG1 was detected with goat anti-human IgG (Fc-specific) conjugated to PO (Jackson Immuno Research) and visualized with TMB (Invitrogen). The TMB reaction was stopped with 1 M HCl after appropriate incubation times at room temperature in the dark. Absorbance was read at 450 nm.

Competition for antigen binding between 2.44IgG1 and the three original murine IgGs was analysed by ELISA as described above, but in this case 500 ng/ml 2.44IgG1 was concurrently incubated on coated and blocked wells with increasing concentrations of the murine IgGs in duplicate and detected with goat anti-human IgG (Fc-specific) conjugated to PO (Jackson Immuno Research).

### Immunofluorescence assay (IFA)

Antibody binding to MSP4 on cell membranes was analysed by immunofluorescence assay (IFA) as previously described with minor modifications [17]. *Pf* 3D7A schizonts from a permanent culture [24] were enriched by Percoll (GE Healthcare) density centrifugation [25] and allowed to mature into membrane-enclosed merozoites by incubation in the presence of 10 μM E64 (Sigma-Aldrich, Taufkirchen, Germany) [26]. IFAs were carried out using methanol-fixed smears of these E64-treated schizonts on SuperFrost microscope slides (Menzel, Braunschweig, Germany). Schizonts were counterstained with 50 μg/ml polyclonal rabbit anti-AMA1 (Ra-AMA1) [27]. Parasite nuclei were counterstained with 10 μg/ml Hoechst 33342 (Sigma-Aldrich). Ra-AMA1 was visualized with goat anti-rabbit IgG conjugated to AlexaFluor®488 or to AlexaFluor®594 (Dianova, Hamburg, Germany), the murine 2.44IgG (50 μg/ml) was visualized with goat anti-mouse IgG conjugated to AlexaFluor®488 (Invitrogen), and recombinant 2.44IgG1 (50 μg/ml) was visualized with goat anti-human IgG conjugated to Cy3 (Dianova). Slides were mounted in ProLong Antifade Gold (Invitrogen) and analysed using a Leica TCS SP8 Confocal Microscope (Leica Microsystems, Wetzlar, Germany).

### Western blot of merozoite extracts

Free merozoites were obtained by filtration of E64-treated schizonts through a 1.2-μm Acrodisc syringe filter with Versapor membrane (Pall, Dreieich, Germany) and were frozen at −80°C at a concentration of 5 × 10^7^ merozoites/ml. After thawing, 600 μl of the suspension was centrifuged at 16,000 x g for 6 min at room temperature, resuspended in 12 μl PBS, separated by SDS-PAGE under non-reducing conditions and blotted onto a nitrocellulose membrane. The blot membrane was blocked with 2% non-fat milk powder in PBS and incubated for one hour with 4 μg 2.44IgG1. The latter was visualized with a goat anti-human IgG (H + L-specific) antibody conjugated to AP (Promega, Mannheim, Germany). The membrane was washed three times for 10 min in PBS with 0.1% Tween-20 between steps and the signal was detected using NBT/BCIP (Roth, Karlsruhe, Germany).

### Surface plasmon resonance (SPR) spectroscopy

The binding of murine and recombinant derivatives of mAb 2.44 was investigated by SPR spectroscopy using a Biacore T200 instrument (Biacore, GE Healthcare) and CM5-S-Series sensor chips. These were either functionalized in one of two ways: (i) with recombinant Protein A (Sigma-Aldrich) immobilized onto flow cell 2 (Fc-2) by EDC/NHS coupling with 12-min activation/deactivation and 20-min contact time, 250 μg/ml protein A in 10 mM sodium acetate pH 4.25, R_immob_ = 4,600 RU, Fc-1 reference EDC/NHS coupling with 12-min activation/deactivation, or, (ii) with the appropriate reagent from the Mouse Antibody Capture Kit (RaM-Fc, Biacore, GE Healthcare) immobilized onto flow cell 4 (Fc-4) by EDC/NHS coupling with 12-min activation/deactivation and 20-min contact time, 50 μg/ml Ram-Fc in 10 mM sodium acetate pH 5.1, R_immob_ = 11,000 RU, Fc-3 reference EDC/NHS coupling with 12-min activation/deactivation. Between measurements, the surface was regenerated by pulsing for 1 min with 30 mM HCl. Fc-1 and Fc-3 were used as references for blank subtraction.

For kinetic analysis, 320–350 RU of the antibody variants (recombinant and murine 2.44) were captured onto immobilized Protein A or RaM-Fc, respectively, and mE-ERH was injected at different concentrations (275 nM serial 1:3 dilution to 3.4 nM) at a flow rate of 30 μl/min for 180 sec. Dissociation was followed for 450 sec. Between measurements, the surface was regenerated by pulsing for 1 min with 30 mM HCl. Buffer injections were used for double referencing. All measurements were taken at 25°C using HBS-EP (10 mM HEPES, 150 mM NaCl, 3 mM EDTA, 0.005% Tween-20) as the running buffer. Binding curves were evaluated using Biacore T200 Evaluation Software (GE Healthcare).

## Results

### Selection of monoclonal hybridoma cell lines

One polyclonal hybridoma cell line was isolated, showing specificity towards mE-ERH, more specifically towards EGF_*Pf*MSP4. After limiting dilution, three monoclonal hybridoma cell lines specific for EGF_*Pf*MSP4 (2.48, 2.44 and 2.7) were selected (Figure 1D), derived from the polyclonal clone with proven specificity for EGF_*Pf*MSP4.

### V-region rescue and sequence identification

**Figure 2:**
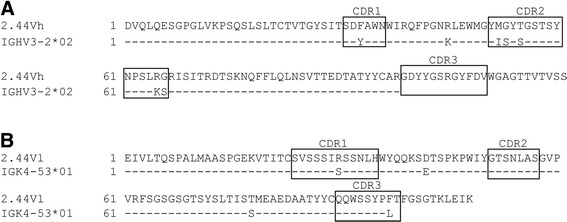
**Sequence alignment of the rescued antibody sequence and the corresponding germ-line sequence.** Antibody V regions were amplified from cDNA isolated from the hybridoma cell line 2.44 using specific primers for the V_H_ and V_L_ regions. The protein-sequence alignment included the most closely related germ-line V gene indicated by the IMGT/V-quest tool. For both, the V_H_ **(A)** and V_L_ **(B)** domains, CDRs 1–3 were determined according to Kabat definitions [51] and are highlighted in boxes.

Total RNA was isolated from the three selected EGF_*Pf*MSP4-specific hybridoma clones, and first-strand cDNA synthesized using oligo-dT primers was used for the specific amplification of V regions. Sequence analysis revealed one unique antibody sequence from all three hybridoma clones, bearing V_H_ and the V_L_ sequences allocated to subtypes V_H_3 and V_L(k)_4. As expected, mutations relative to germ-line sequences were predominantly found within the CDRs and were less prevalent in the FWRs (Figure 2). Unfortunately, the primer set not only amplified the specific PCR product, but also the aberrant Igκ pseudogene from the myeloma SP2/0 cells (using primer 5′L-Vk-3 [8]) but this known sequence [28] was easily identified using NCBI nucleotide BLAST. The functional V_H_ region was thus amplified using the primers 5′-AAC AAC ACC GGT GTA CAT TCC GAT GTG CAG CTT CAG GAG TCG GG-3′ and 5′-GTT GTT GTC GAC GCT GAG GAG ACG GTG ACC GTG GTC CC-3′. Accordingly, the functional V_L(κ)_ sequence was amplified using the primers 5′-AAC TGC AAC CGG TGT ACA TTC CGA AAT TGT CCT CAC CCA GTC TCC-3′ and 5′-GTT GTT CGT ACG TTT TAT TTC CAA CTT TGT CCC C-3′.

### Cloning, expression and binding analysis of recombinant 2.44IgG1

**Figure 3:**
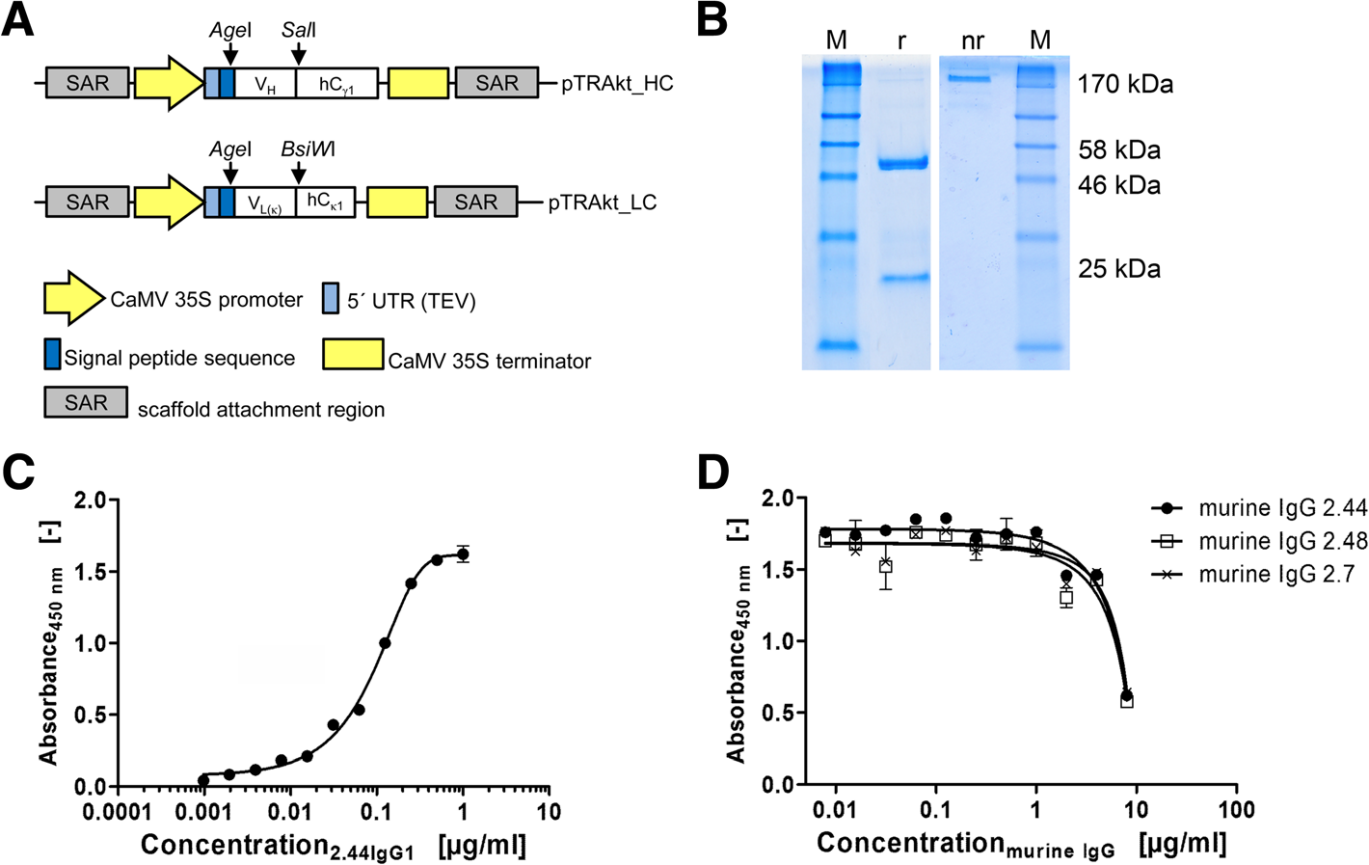
**Generation and characterization of recombinant 2.44IgG1. (A)** Expression cassette of pTRAkt_HC and pTRAkt_LC. The vectors contain the CaMV 35S promoter , a 5′ UTR of the TEV (5′ UTR (TEV)) and a murine IgG signal sequence targeting the apoplast Two vectors were constructed, one containing the genetic information for the hC_γ1_ sequence (pTRAkt_HC) and one containing the genetic information for the hC_κ1_ sequence (pTRAkt_LC). The rescued V_H_ and V_L_ regions were inserted using the *Age*I/*Sal*I (V_H_) or *Age*I/*BsiW*I (V_L_) restriction sites. **(B)** Purity and assembly of recombinant 2.44IgG1. Recombinant 2.44IgG1 was purified from filtered plant extract by MabSelect^TM^ chromatography, and 2 μg of purified 2.44IgG1 was analysed for purity and correct antibody assembly by SDS-PAGE (12% polyacrylamide) under non-reducing (nr) and reducing (r) conditions. **(C)** Recognition of mE-ERH by recombinant 2.44IgG1. The antigen-binding activity of recombinant 2.44IgG1 was analysed by ELISA using 100 ng mE-ERH per well. Recombinant 2.44IgG1 was probed with goat anti-human IgG_Fc_^PO^, visualized with TMB whose reaction was stopped with 1 M HCl. **(D)** Competition of recombinant 2.44IgG1 by parental murine IgGs. To compare the binding activity of the recombinant antibody and the three murine antibodies, a competition ELISA was carried out using a constant concentration of 500 ng/ml recombinant 2.44IgG1 and increasing concentrations of the murine antibodies. Recombinant 2.44IgG1 was probed with goat anti-human IgG_Fc_^PO^, visualized with TMB whose reaction was stopped with 1 M HCl.

The V regions were transferred into the respective pTRAkt expression vectors (Figure 3A) and introduced into electrocompetent *Agrobacterium tumefaciens*, which were subsequently used for the infiltration of *Nicotiana benthamiana* plants. Following transient expression, the recombinant 2.44IgG1 was successfully purified by MabSelect™ chromatography, yielding 45 mg of antibody per kg of fresh leaf weight (Figure 3B). Specific binding to mE-ERH was confirmed by ELISA (Figure 3C). Specific antigen binding, defined as > three-fold background absorbance at 450 nm (0.045 units), was detectable at antibody concentrations as low as 8 ng/ml. Using this setup, saturation of the antibody occurred at a concentration of 500 ng/ml.

A competition ELISA was used to compare the epitope of the initial hybridoma antibodies and the recombinant derivative. Antigen binding was equally inhibited by all three hybridoma cell lines, confirming the identity of these three clones (Figure 3D). The affinities of the murine and recombinant antibodies were determined by SPR spectroscopy, revealing similar affinity constants (K_D_) of 8 nM (±0.15 nM) and 10 nM (±0.01 nM) for the parental murine antibody and the recombinant antibody, respectively (Table 1).

**Table 1:** **Affinities of the murine and the recombinant 2.44IgG1 mAb determined by SPR spectroscopy**

**Antibody**	**k**_**a**_ **(SE) [1/Ms]**	**k**_**d**_ **(SE) [1/s]**	**K**_**D**_ **(SE) [M]**
Murine 2.44	7.18E + 04 (3.10E + 02)	5.74E-04 (8.2E-06)	8.00E-09 (1.49E-10)
Recombinant 2.44	9.04E + 04 (3.3E + 01)	9.26E-04 (7.1E-07)	1.02E-08 (1.16E-11)

k_a_ = association constant; k_d_ = dissociation constant; K_D_ = affinity constant = k_d_/k_a_.

SE = standard error.

### Detection of native PfSMP4 by murine and recombinant 2.44IgG1

**Figure 4:**
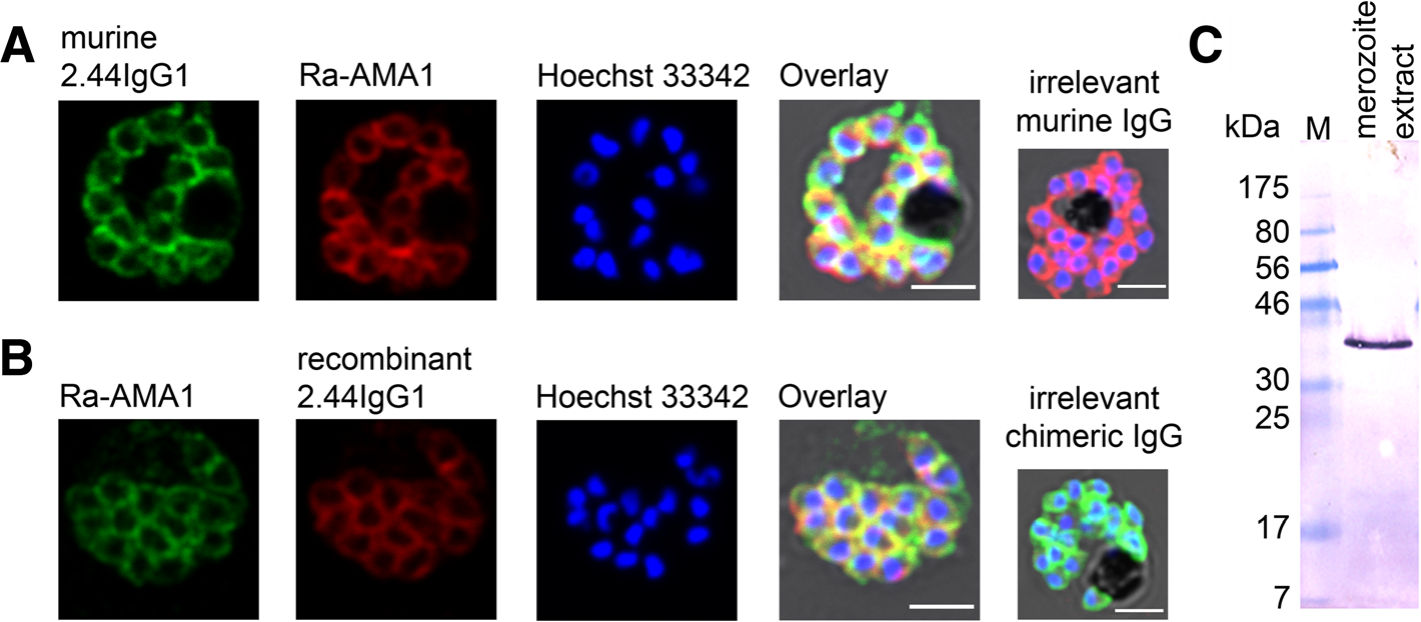
**Detection of native** ***Pf*** **MSP4 by recombinant 2.44IgG1. (A)** Specific binding of the murine 2.44IgG1 to methanol-fixed *Pf* 3D7A schizonts was assessed by IFA. The murine 2.44IgG1 was visualized with AlexaFluor®488-conjugated goat anti-mouse IgG secondary antibody (green) and AMA1 on the parasite surface was stained with Ra-AMA1 which was visualized with AlexaFluor®594 conjugated secondary antibody (red), showing the circular localization of AMA1 in mature schizonts. Nuclei were stained with Hoechst 33342 (blue). The overlay image includes the bright field image. The small overlay shows the lack of green staining obtained with an irrelevant murine IgG. Scale bar = 2.5 μm. **(B)** AMA1 on the parasite surface was stained with Ra-AMA1 and visualized with AlexaFluor®488-conjugated secondary antibody (green), showing a circular localization of AMA1 indicating fully mature schizonts. The recombinant 2.44IgG1 was visualized with a Cy3-conjugated goat anti-human IgG secondary antibody (red). Nuclei were stained with Hoechst 33342 (blue). The overlay image includes the bright field image. The small overlay shows the lack of red staining obtained with an irrelevant chimeric IgG. Scale bar = 2.5 μm. **(C)** Detection of *Pf*MSP4 by recombinant 2.44IgG1 using western blot analysis. A western blot of SDS-PAGE-separated merozoite extract was probed with 4 μg of the recombinant 2.44IgG1 revealing one specific band (~40 kDa), indicated with an arrow, which corresponds to the anticipated molecular size of *Pf*MSP4.

IFAs using synchronous *Pf* 3D7A parasites were performed to test the ability of antibodies to bind to the GPI-anchored MSP4 protein, which is homogenously distributed on the merozoite surface. The immunofluorescence signal confirmed the staining of merozoite surface proteins at the late schizont stage (Figure 4A and B), similar to the AMA1 staining pattern at this stage. IFAs using the murine antibody and the recombinant antibody showed the same staining pattern. Unspecific binding to other MSPs was excluded by the detection of a single 40-kDa band in merozoite extracts by western blot, corresponding to the anticipated molecular size of MSP4 (Figure 4C) [29].

## Discussion

The here-applied strategy for the rapid and accurate rescue of V-region sequences was adapted to the strategy which was introduced by Tiller *et al*. [8,30]. Major alterations in the method presented in this work are the design of the V_H_-region forward primer, the introduction of *Nicotiana benthamiana* as the expression host and the application of analytical methods, which are of essential importance for the characterization of antibodies specific for *P. falciparum*. A mouse (V region)-human (Fc region) chimeric mAb was generated instead of reconstituting a murine full size IgG, because these partially humanized antibodies are usable in other assays investigating the cellular participation in the antimalarial immune response, such as ADCI or ADRB [31,32].

The combination of these methods is suitable for routine use in any laboratory familiar with hybridoma cell lines, and can be used to eliminate the risk of losing the genetic information of unique antibodies. V-region rescue and sequence analysis is helpful to avoid working with apparently different clones producing identical mAbs as a result of clone proliferation during the limiting dilution steps used in the hybridoma generation [33]. Amplifying the V regions of antibodies starting off with mRNA isolation from hybridoma cells can be a labour-intensive process, but the here-used method reduces the workload to a minimum while ensuring that V-region sequences are rescued with high efficiency and accuracy, because as reported by Tiller *et al*., the primer set achieves ~60% rescue efficiency in a single cell PCR method, and all highly abundant V-gene families are amplified [8]. Therefore, it can be expected that the rescue efficiency based on cDNA from a larger amount of cells will be significantly higher.

In contrast to other primer sets that are entirely based on primers containing degenerate and/or consensus positions, the here-used primer set reduces the risk of primer-derived mutations to a minimum since only the consensus forward primer of the V_H_ region contains such sequence ambiguity. Sequence mutations have to be avoided, because even one single substitution within antibody FWRs may reduce or even completely abolish antigen binding [34]. De Haard *et al*. identified residue 6 (FWR1) of the V_H_ region as one of the most crucial residues for correct antibody folding and thus antigen recognition. It is, therefore, important to verify that the rescued sequence truly represents genetic information that determines the affinity of the corresponding antibody. An alternative approach to confirm the rescued V_H_-sequence is the use of degenerate primers in combination with N-terminal peptide sequencing of the original murine antibody [35]. This method is feasible, but more expensive and often not directly available. Therefore, the rescued sequence information was analysed by comparison with the open-access online database IMGT, but also here alternatives are available such as VBASE2 and NCBI [20,36,37]. All of these have assembled comprehensive antibody germ-line sequence data. V_H_-FWR1 residues could be verified to match the corresponding germ-line sequence, which generally provides the best guess regarding the respective residue. Although that at least in the human system V_H_-FWR1 mutations can occur in heavily affinity matured antibodies even at the highly conserved position 6, the V_H_-FWR1 region is the region which seems to be moderately affected by somatic hypermutation when compared to other regions [38,39]. Additionally to the here-presented EGF_*Pf*MSP4-specific antibody, this technique was successfully applied to rescue V-region sequences from several anti-malarial murine mAbs, including mAbs against AMA1, MSP3 and HRP2 (unpublished data).

Furthermore, the rescued sequences can be used to generate any recombinant antibody format, including scFv, Fab, F(ab)_2_ and full-size antibodies featuring Fc-regions of the required species and isotype. Even though these formats can be generated more quickly if the confirmation of the V-region rescue by the here-suggested analyses is omitted, but the results may be less satisfactory, since uncorrected primer-derived mutations may lead to loss of epitope-specificity and affinity as well as problems with expression yields and solubility [12]. The analysis of recombinant mAbs based on the rescued V regions circumvents these drawbacks by allowing the direct comparison of the antigen-binding properties of the rescued antibody and its original hybridoma-produced counterpart. Additionally, the recombinant format of the antibody allows a completely new therapeutic approach in the malaria field, anti-malarial antibody fusion proteins. In such an antibody fusion protein, an scFv is genetically fused to a protein with anti-malarial activity. The resulting protein has a significantly increased activity and therefore meets the criteria for a potential therapeutic agent [40].

The here-applied plant-based transient expression of a mouse-human chimeric full-size antibody has been shown to yield a functional mAb at reduced costs and saved time [41]. V-region sequences can be cloned into appropriate vectors, expressed transiently in *Nicotiana benthamiana* by agro-infiltration and purified within two to three weeks after sequence rescue and analysis, in this case yielding >40 mg/kg of pure full-size IgGs with similar binding characteristics as shown by SPR spectroscopy analysis. In mammalian expression systems, very high yields are only achieved after cost- and time-consuming cell line development and carefully regulated cultivation of cells [42]. Furthermore, the agro-infiltration-based transient plant expression system provides the opportunity to easily express different recombinant proteins (e.g., heavy and light chains of antibodies) simultaneously by co-infiltration. Different leaves of the same plant can be used to efficiently compare independent mAbs as well as different combinations of antibody heavy and light chains or mutants thereof, making this system ideally suited for the time-efficient production and analysis of antibody sequences derived from a hybridoma antibody cloning approach.

In the context of malaria research, antibodies can be used for the functional analysis of potential vaccine targets, as well as therapeutic molecules. Besides the determination of epitopes and antigen-binding characteristics, the analysis of parasite growth inhibitory efficacy in various *in vitro* assays, including the blood-stage growth inhibition assay (GIA), transmission blocking assays (TBA) or the inhibition of sporozoite invasion (ISI), is essential to asses the suitability of a mAb for the desired application. Therefore it is beneficial if the mAbs can be efficiently produced at mg quantities at low cost in one single batch to facilitate the concurrent screening of several mAbs with potential for the desired downstream application. The purified recombinant mAb can directly be compared with the parental murine antibody by appropriate analytical methods like competition ELISA and/or SPR spectroscopy-based affinity determination, to confirm the rescue of the correct V regions and thus avoid loss of the antibody if the original hybridoma cell line becomes unstable or dies off.

If assays including cellular immune responses are the objective of further investigations besides blocking activity of the mAb, as it is the case in GIAs, TBAs and ISIs, several aspects still need to be considered. It has been shown that murine mAbs were successful in protecting mice against parasite-challenge, but their corresponding chimeric mAbs (bearing human Fc regions) were not [43]. In contrast, when mice were transfected with the human receptor I for IgG (FcγRI, CD64), fully human mAbs derived from semi-immune Gambian adults were able to control parasitemia [44]. This indicates the importance of the IgG-Fc region for protection in its parental species. For human applications, fully human mAbs have the significant advantage of not inducing any human anti-mouse immune responses, like fully murine, and also mouse-human chimeric mAbs do, as introduced in the background section. Therefore, a homologous workflow for the generation of fully human antibodies is currently being set up at the Fraunhofer-Institute for Molecular Biology and Applied Ecology IME (Aachen, Germany) [45].

At this point, it has to be mentioned that the IgG-Fc N-glycan profile essentially contributes to FcγR engagement [46], which is crucial for the effector cell recruitment *in vivo* and similarly for the respective assays *in vitro* (ADCI, ADRB). Plant-derived mAbs bear a distinct IgG-Fc N-glycan profile, and especially the plant-specific β1-2 xylose located at the bisecting mannose has been shown to interfere with binding to IgG-FcRs (FcγRs) [47]. If these applications are finally striven for, either a mammalian expression system may be chosen, or genetically modified plants which attach a humanized IgG-Fc N-glycan profile. The knockdown of the xylosetransferase-gene has been shown to restore binding of a plant-derived mAb to its FcγR [47].

It has recently been discussed, whether plant-derived mAbs are applicable acute-phase drugs, especially against the viral disease Ebola, one of the major acute burdens in Western Africa [48,49]. The major obstacle here is the impressively large scale of ~100 kg plant material to yield one complete dose (10.5 g) for human application [48]. One licensed therapeutic antibody against CD20-positive lymphoma, rituximab, requires an annual dose of ~1.8 g per patient [50], somewhat similar to the estimated dose required to efficiently control parasitemia in FcγRI-transgenic mice [44] (1.5 mg mAb/mouse, adding up to ~2.6 g per human of 70 kg, resulting in 25 kg plant material, given an expression rate of 100 mg mAb/kg fresh leaf weight, according to the calculations by Rybicki [48]). Finally, the work here describes a simple and fast work flow for the initial screening of a mAb’s characteristics and functionality. Indisputable, there is still many research to be done to meet the criteria for a cost-efficient production of a final, especially mAb-based, therapeutic against poverty-related diseases, such as ebola, or malaria.

## Conclusions

Antibody V-region rescue is an essential technique to preserve the unique epitope binding specificity of mAbs. Here, an efficient combination of methods for the rescue and transient plant-based expression of murine antibody sequences is presented, using an EGF_*Pf*MSP4-specific antibody as an example. The complete workflow is described in detail, including initial V-region amplification, cloning, heterologous expression in *Nicotiana benthamiana* and verification of the rescued sequences by comparative functional analyses. The described method can be applied to facilitate the rescue, production and characterization of larger numbers of monoclonal antibodies. This is especially relevant in the field of malaria research where the plurality of *P. falciparum* proteins makes it necessary for investigators to create their own specific mAbs for analytical purposes, to investigate new vaccine targets or as starting point for antibody-based therapeutics.

## Abbreviations

ABTS: 2,2′-azino-bis(3-ethylbenzothiazoline-6-sulphonic acid)
AP: Alkaline phosphatase
C1-3: Constant regions 1–3
cDNA: complementary DNA
CDR: Complementarity determining region
EGF: Epidermal growth factor
EGF_*Pf*MSP4: EGF-like domain of *P. falciparum* MSP4
ELISA: Enzyme linked immunosorbent assay
FcγR: Fc-receptor for IgG
FWR: Framework region
GPI: Glycophosphatidylinositol
IFA: Immunofluorescence assay
mAb: monoclonal antibody
mE-ERH: Fusion protein containing the EGF-like domains of MSP1-19, MSP8 and MSP4
MSP: Merozoite surface protein
NBT/BCIP: Nitroblue tetrazolium chloride/5-bromo-4-chloro-3-indolyphosphate p-toluidine salt
PBS: Phosphate-buffered saline
PBS-T: Phosphate buffered saline with 0.1% Tween-20
PCR: Polymerase chain reaction
PO: Peroxidase
scFv: single chain fragment variable
SDS-PAGE: Sodium dodecylsulphate polyacrylamide gel electrophoreses
SPR: Surface plasmon resonance
TEV: Tobacco Etch Virus
TMB: 3,3′,5,5′-tetramethylbenzidine
UTR: Untranslated region
V_H/L_ region: Variable region of the antibody heavy or light chain
